# Dual functional dinuclear platinum complex with selective reactivity towards c-myc G-quadruplex

**DOI:** 10.1038/s41598-017-19095-y

**Published:** 2018-01-15

**Authors:** Lei He, Zhenyu Meng, Dechen Xu, Fangwei Shao

**Affiliations:** Division of Chemistry and Biological Chemistry, School of Physical and Mathematical Sciences, Nanyang Technological University, 21 Nanyang Link, Singapore, 637371 Singapore

## Abstract

G-quadruplexes (GQ) folded by the oncogenic G-rich sequences are the promising targets for developing anticancer therapeutic molecules. However, the current drug development mainly focused on non-covalent dynamic binders to stabilize GQ structures, while the covalent targeting from inorganic complexes *via* chelating principles, as a potent therapeutic strategy was surprisingly lack of exploration. Herein, a series of dinuclear platinum complexes, [(Pt(Dip)Cl)_2_(μ-diamine)](NO_3_)_2_ (Dip: 4,7-diphenyl-1,10-phenanthroline), were designed to contain two dual-functional Pt cores connected by an alkyl linkage. Pt3 with nonanediamine linkage optimized the specific binding towards c-myc G-quadruplex *via* dual functional clamp on GQ as 1) non-covalently π-stacking of aromatic ligands, and 2) two Pt(II) cores covalently chelated to guanines at both 3′- and 5′-ends.

## Introduction

Guanine-rich DNA sequences are prone to self-assemble into several layers of planar G-tetrad by Hoogsteen hydrogen bonds in the presence of cations, such as sodium and potassium ion. Stacking of more than two layers of G-tetrads provided guanine quadruplexes (GQ)^1–4^. GQ was folded by the G-rich sequences from human telomeres that located at the terminal of chromosomes, as well as the promotor regions of many oncogenes, such as c-myc, c-kit and bcl2^5–7^. The formation of G-quadruplexes was proposed to either regulate the oncogene expression or inhibit the telomerase activity^8–10^.

The significant biological roles of GQ attracted great interests to design GQ-targeting molecules for cancer therapy. So far, substantial amount of organic compounds and metal complexes have been reported with excellent stabilization on GQ mainly *via* π-π stacking with G-tetrads and lead to potent antitumor activity^11–14^. Among these binders, dinuclear metal complexes, due to the unique cooperative bindings of two complex components, achieved excellent selectivity for GQ structures and the reduced drug tolerance of tumour cells comparing to mononuclear metal complexes as anticancer drug candidates^15–17^. Beyond non-covalent interactions with DNA, Pt(II) complexes can covalently cross-link to the purines. Pt-DNA adducts could interfere the recognition/binding of protein factors and the downstream biological pathways to exert the anticancer therapeutic effects. For example, cisplatin formed an intra-strand adduct on telomere sequences, which inhibited telomerase activity and, eventually induced telomere loss and tumour cell death^18,19^. Bombard and co-workers demonstrated the cross-linking between human telomere sequence and cis-/transplatin, with several binding sites including adenines in the loop region and guanines in the external G-quartet^20^. Though small molecules as both GQ stabilizer and cross-linking agents could be more promising in anticancer potency, the studies of these dual functional drug candidates are significantly inadequate. A couple of organic molecules as GQ stabilizer were conjugated with monochloro-platinum complexes to achieve a dual noncovalent/covalent interaction with telomeric GQ^21,22^. Teulade-Fichou group showed a set of mononuclear Pt-terpyridine complexes could exclusively coordinate to the loop adenine, meanwhile, with an enhanced binding affinity and platinating activity after extending the aromatic surface of the terpyridine moiety^23^. Further modification of Pt-tolylterpyridine complex by classical photo-crosslinking groups-benzophenone and tetraphenylazide can achieved improved selectivity to different GQ sequences, with the formation of a second covalent bond between Pt-ttpy-N_3_ and GQ upon photoactivation^24^. Whereas, dinuclear metal complex possessed unique binding module on GQ and is a good means to offer a novel approach to form dual functions of both long-range cross-linking and stabilization on G-tetrads.

**Figure 1:**
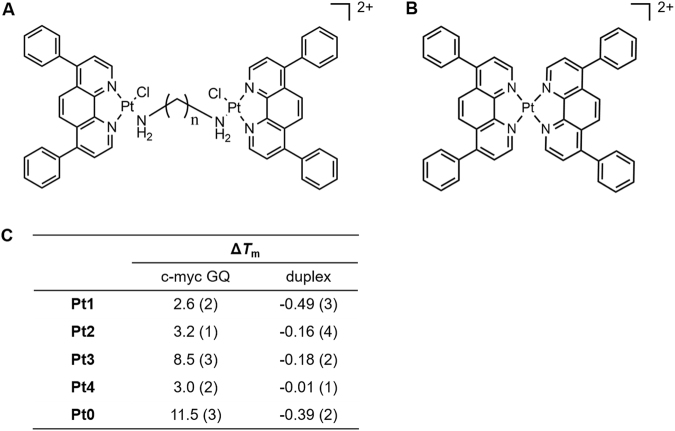
(**A**) Structures of dinuclear platinum complexes **Pt1-Pt4**, n = 7–10. (**B**) Structure of **Pt0**. (**C**) Enhancements of melting temperatures (Δ*T*_m_) of c-myc GQ and duplex DNA in the presences of dinuclear Pt complexes and **Pt0**. Error bars on last significant digit are listed in parentheses.

Our group has recently reported a platinum compound, [Pt(Dip)_2_](PF_6_)_2_ (**Pt0**, Dip: 4,7-diphenyl-1,10-phenanthroline, Fig. 1), which can efficiently stabilize GQ with aromatic stacking of phenanthroline ligands and further possess high binding preference towards parallel c-myc GQ *via* four phenyl groups docking into the lateral grooves^25^. In order to achieve the dual stabilization and cross-linking functions, we designed and synthesized a series of dinuclear Pt-Dip compounds (Fig. 1A) with the following properties: (1) each Pt(II) coordinated to a Dip ligand in order to offer π-π stacking on G-tetrads; (2) One chloride was chelated to each Pt(II) and acted as a leaving group to enable the cross-linking reaction to the purine bases in GQ sequences, such as guanine; (3) An alkyl diamine filled the last coordination site to link two [Pt(Dip)Cl] components. Alkyl linkages with seven to ten methylene groups were used to optimize the distances between two complex components for simultaneous non-covalent and covalent interactions with GQ structures.

## Results

### Design and synthesis of the dinuclear platinum complexes

Dinuclear platinum complex **Pt1** to **Pt4** were synthesized as Scheme S1. Pt(Dip)Cl_2_ was prepared as literature^25^. Silver nitrate was first used to remove one chloride from Pt(Dip)Cl_2_ which enabled alkyl diamine to connect two Pt centres *via* coordination of amine. The lengths of the alkyl chain were ranged from 7.7~11.5 Å, while the rise of three layers of G-tetrads was around 10 Å. The flexible alkyl chain would place the two Pt complexes within a distance of ±2 Å around the height of the aromatic core in c-myc GQ. Characterizations of these dinuclear platinum complexes were performed by mass spectrometry and ^1^H NMR (Figure S1). Only one set of aromatic protons was observed in the spectrum, which was consistent with the symmetry of the dinuclear platinum compounds. Due to the high flexibility of alkyl chain, crystallization of Pt3 was not successful and the structure of dinuclear Pt3 complex was further confirmed by 2D ^1^H-^1^H COSY and ROESY NMR spectra (Figure S2). Off diagonal peaks superimposed in COSY and ROESY spectra indicated that aromatic protons on Dip ligands were in close vicinity of aliphatic protons in alkyl linkage.

### Binding and stabilizing selectivity of **Pt3** to c-myc GQ

Thermal stabilization effects of the dinuclear Pt complexes on different DNA structures were explored by using FRET melting assays. As shown in Figure S3 and Table S1, upon addition of one equivalent amount of each Pt complex, the melting temperature (Δ*T*_m_) of duplex DNA showed no increment above error bar, while G-quadruplexes with variety of folding topology showed small to mild Δ*T*_m_ enhancement (0.4~8.5 °C) in the presence of different Pt complexes. Furthermore, among all the GQ topology examined, highest Δ*T*_m_ always occurred to c-myc GQ regardless of dinuclear Pt complexes. The similar topological selectivity towards parallel c-myc GQ as mononuclear **Pt0** suggested that Pt(Dip) component in dinuclear complexes possessed the similar stacking mode as that of **Pt0**. **Pt3** showed the highest Δ*T*_m_ on c-myc GQ (8.5 °C) among all the Pt compounds (2.6, 3.2 and 3.0 °C for **Pt1**, **2** and **4**, respectively) (Fig. 1C), though *T*_m_ elevation on c-myc GQ was a little lower than **Pt0** owing to the lack of second Dip ligand coordinated to the same Pt centre. The enhanced thermal stability suggested the length of alkyl linkage in **Pt3** was optimal for the two Pt(Dip) components to achieve stable interactions with c-myc GQ. Moreover, competitive FRET-melting experiment was carried out with c-myc GQ in the presence of an excess ds26. As shown in Figure S4, melting temperature of c-myc GQ in the presence of **Pt3** showed no significant attenuation even in the presence of 50 equivalents of duplex DNA, which further showed the selective binding of **Pt3** towards parallel GQ over over duplexes. The selectivity of **Pt3** towards c-myc GQ was further investigated by fluorescent intercalator displacement (FID) assay (Figure S5). Thiazole orange (TO) interacted with GQ by stacking with G-tetrads or binding to the grooves/loops. The submicromolar displacement efficiency of **Pt3** on c-myc GQ (DC_50_ was 0.20 μM) was nearly 6 and 10 folds higher than that on telomeric GQ (ht21) and duplex DNA (ds26), respectively, which further suggested the higher binding selectivity of **Pt3** for c-myc GQ (Table S2).

### **Pt3** selectively dual cross-linked to c-myc GQ

**Figure 2:**
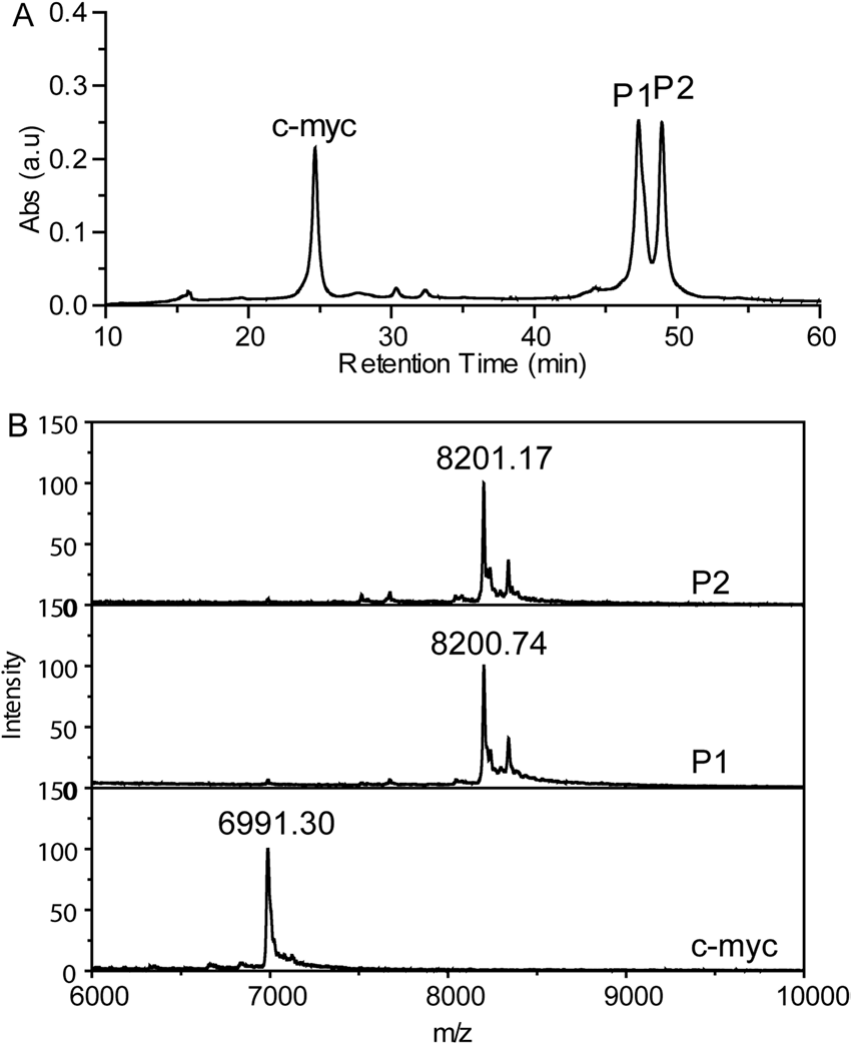
(**A**) HPLC analysis of the crudes from crosslinking reaction between **Pt3** and c-myc GQ and (**B**) MALDI-TOF mass spectra of free c-myc GQ and the **Pt3**-GQ adducts, **P1** and **P2**.

Due to the optimal stabilizing effects on c-myc GQ, **Pt3** was used for the study of the cross-linking reactions to GQ. The cross-linking reactions were performed in potassium phosphate buffer with high concentration of KNO_3_ (100 mM), to keep the folding structure of c-myc GQ and avoid the free Cl^−^ ions binding back to Pt center, which will influence on the cross-linking. Both c-myc and ht21 GQ in both buffers folded into the parallel and antiparallel topology, respectively, since their CD spectra showed the respective characteristic peaks with the same intensity (Figure S6). Remarkably, **Pt3** formed cross-linking adducts with c-myc GQ efficiently. As shown in Fig. 2A, 76% of c-myc GQ reacted with **Pt3** after incubation at 37 °C for 24 hours, which yielded two new products, **P1** and **P2**, with longer retention time of around 47 minutes on HPLC analysis. In contrast to c-myc GQ, it is noteworthy that no new peak emerged upon HPLC analysis of the same platination reactions with ht21 GQ (Figure S7). Almost no reactivity with telomeric GQ indicated that **Pt3** also held high selectivity to parallel GQ topology in the cross-linking reactions. In the case of ds26, only less than 10% duplex DNA was cross-linking to **Pt3** (Figure S8 and Table S3). Such low crosslinking yields occurred to both antiparallel GQ and duplex DNA may be due to the binding selectivity of **Pt3** to c-myc GQ over other DNA structures and/or better accessibility of purines in c-myc GQ for cross-linking reactions. Furthermore, upon incubating the other three Pt complexes with c-myc GQ, the reaction mix showed no peaks at the retention of cross-linking adducts. Different from **Pt3**, **Pt1**, **2** and **4** showed no cross-linking reactivity towards c-myc GQ, which is presumably due to their weak binding to c-myc GQ (Figure S9). MALDI-TOF mass spectrometry was further used to characterize the exact mass of two **Pt3**-GQ adducts, **P1** and **P2**, that were collected from HPLC purification. MALDI-TOF results for both **P1** and **P2** showed the molecular weight of a dual cross-linking product (M.W. = 8200.7 obsd. 8201.8 calc.). This M.W. was equal to **Pt3**-**2Cl** (**Pt3** minus two Cl: [C57H54N6Pt2] = 1209.4), plus c-myc GQ ([C220H270N95O131P21] = 6991.2) and indicated that one cross-linking bond was formed between one purine and each Pt(II) center (Fig. 2B and Table S4). However, the distinct retention times upon HPLC separation suggested that two adducts may have discrepancy on either cross-linking sites or the topological conformation, though they showed the identical molecular mass.

### **Pt3** cross-linked to 3′ and 5′-end guanines on c-myc GQ

**Figure 3:**
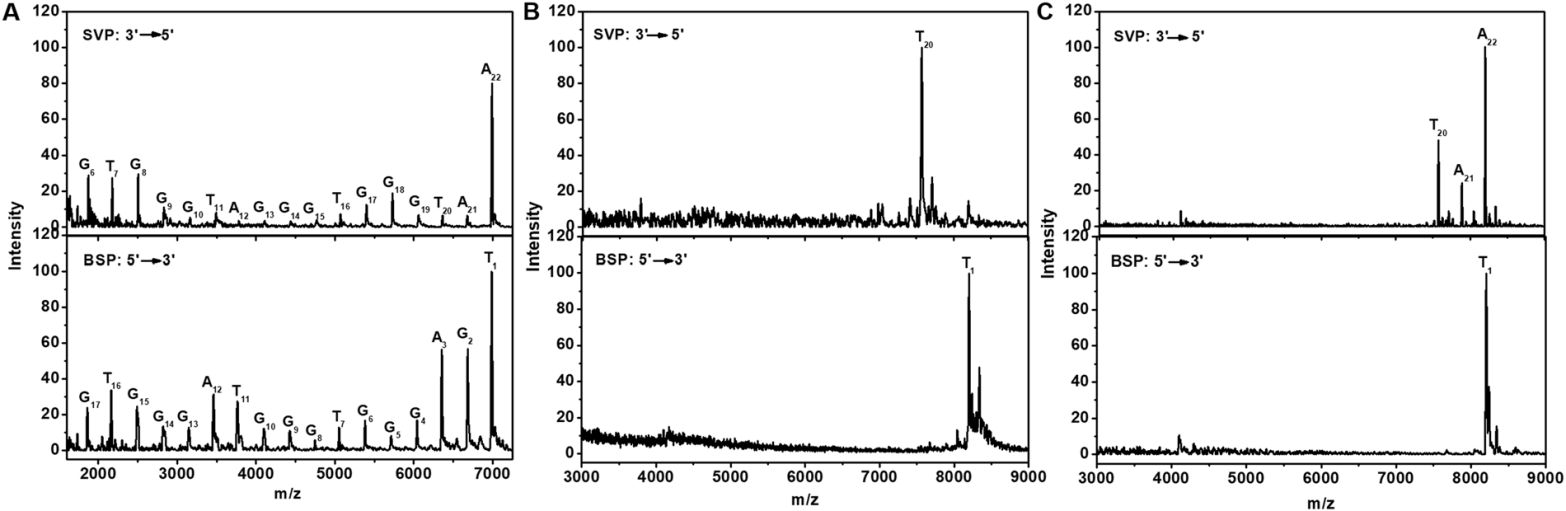
MALDI-TOF mass spectra of (**A**) c-myc and (**B** and **C**) **Pt3**-GQ adducts (**P1** and **P2**) after enzyme digestion.

There are a total of thirteen guanines and four adenines in c-myc GQ sequence. In order to identify the potential cross-linking sites, mass spectrometry was firstly applied to explore the reactivity of adenosine and guanosine with **Pt3**. As shown in Figure S10, after 24 hours reaction, all **Pt3** formed **Pt3**-rG adducts after incubating with guanosine and molecular mass for [Pt(Dip)(rG)] (M.W. = 809.33) and for [{Pt(Dip)(dG)}_2_(μ-1,9-diaminonoane)]^2+^ (M.W. = 888.85) were found. Whereas, the same reaction with adenosine only yield negligible amount of **Pt3**-rA adducts (M.W. = 793.21 for [Pt(Dip)(rA)]; M.W. = 829.11 for [Pt(Dip)Cl(rA)]). The crosslinking reaction only occurred to rG when **Pt3** was treated with a mixture of the same amount of rA and rG. Mass spectrum suggested that the dinuclear **Pt3** had much higher intrinsic reactivity to guanine than adenine, if not exclusive reactivity. Considering the accessibility of N7 from purines within a GQ structure could be modified, a protocol of enzymatic digestion accompanied with MALDI-TOF mass characterization was further developed to allocate the crosslinking sites in c-myc GQ. Two exonucleases, snake venom phosphodiesterase (SVP) and bovine spleen phosphodiesterase (BSP), were used to cleave c-myc GQ at one nucleotides per step from 3′- and 5′-ends, respectively^26–28^. Since the cross-linking to Pt complex could stall the enzymatic cleavage, the highest mass peak from the digestion products of **Pt3**-GQ adducts can be used to identify the first covalent binding sites from 3′- and 5′-ends of c-myc GQ. Under the enzymatic protocols of two exonucleases, partial digestions of free c-myc GQ was performed in order to yield both molecular mass of entire G-rich sequence and c-myc fragments for the sequencing purposes by MALDI-TOF mass spectrometer (Fig. 3A). The partial digestion was used in purpose to avoid the bypass of platination sites in **Pt3**-GQ adducts under excess enzymatic degradation^20^. To avoid the low digest efficiency due to the tight folding structure of G-quadruplex, which reduced the accessibility and process of the enzymes, all the samples were subjected to thermal denature before the enzymatic sequencing protocol. Unlike free c-myc GQ, both **Pt3**-GQ adducts showed much fewer cleavage products (Fig. 3B and C). BSP activity on the 5′-end of GQ sequence was completely inhibited and both adducts, **P1** and **P2**, yielded only molecular weight of dual cross-linking **Pt3**-GQ complex on mass spectra. This indicated that G_2_ was the crosslinking site to one Pt(Dip) complex in both adducts. Upon digestion by SVP, cleavage up to T_20_ from 3′-end of **P1** and **P2** was observed, which indicated that 3′-exonuclease was stalled after cleavage of two adenines, A_21_ and A_22_. The similar mass spectra of enzymatic products from **P1** and **P2** suggested that the same cross-linking sites between **Pt3** and c-myc GQ occurred in both adducts. One Pt nuclei coordinated to G_2_, while the second Pt core chelated to G_19_, which indicated that the guanine that involved in the external G-quartet formation can be platinated by **Pt3**. This similar conjugating disturbance to G-quartet was also observed in the Pt-adducts of human telomeric GQ^29^. Both cross-linking bonds prohibited the enzymatic cleavage of the phosphodiester linkage between the reactive guanines and the neighbouring thymines. However, the enzymatic activity of SVP showed slightly different efficiency on **P1** and **P2**. Only one fragment peak was observed after SVP treatment on **P1**, indicating the adduct had completely lost the 3′-end adenines. Whereas, larger mass including molecular weight of **P2** (8201.2) and **P2**-A_22_ (7887.5) were also detected after the same enzymatic treatment was applied on **P2**. The discrepancy in SVP reactivity suggested that **P1** and **P2** may stay at distinct conformation that resulted the variation in 3′-end accessibility to exonucleases, though both adducts contained the cross-linking bonds to the same guanines.

### Two conformations of **Pt3**-c-myc GQ adducts provided by computational simulation

The possible structures of **Pt3**-GQ adducts were further explored by computational simulation. The molecular structure of **Pt3** was optimized and docked onto c-myc GQ (PDB ID: 2L7V) by GOLD. To find the possible binding conformation of **Pt3** for dual cross-linking reactions, a constraint force between two Pt atoms and N7 of G_2_ and G_19_ was applied for docking, respectively. Two binding structures with highest docking scores and hence energy minimum were obtained (Figure S11)^30,31^. For both conformations, one Pt(Dip) complex fitted into the 5′-end of c-myc GQ with the phenanthroline plane stacking onto the bottom G-tetrad, while the second Pt core resided near the 3′-end of the G-quadruplex. The alkyl linkage connecting the two Pt(II) nuclei, either stretched across the backbone of G_17_ in the first conformation (Conf 1) or resided along the groove between the first and last G-triplet in the second conformation (Conf 2).

**Figure 4:**
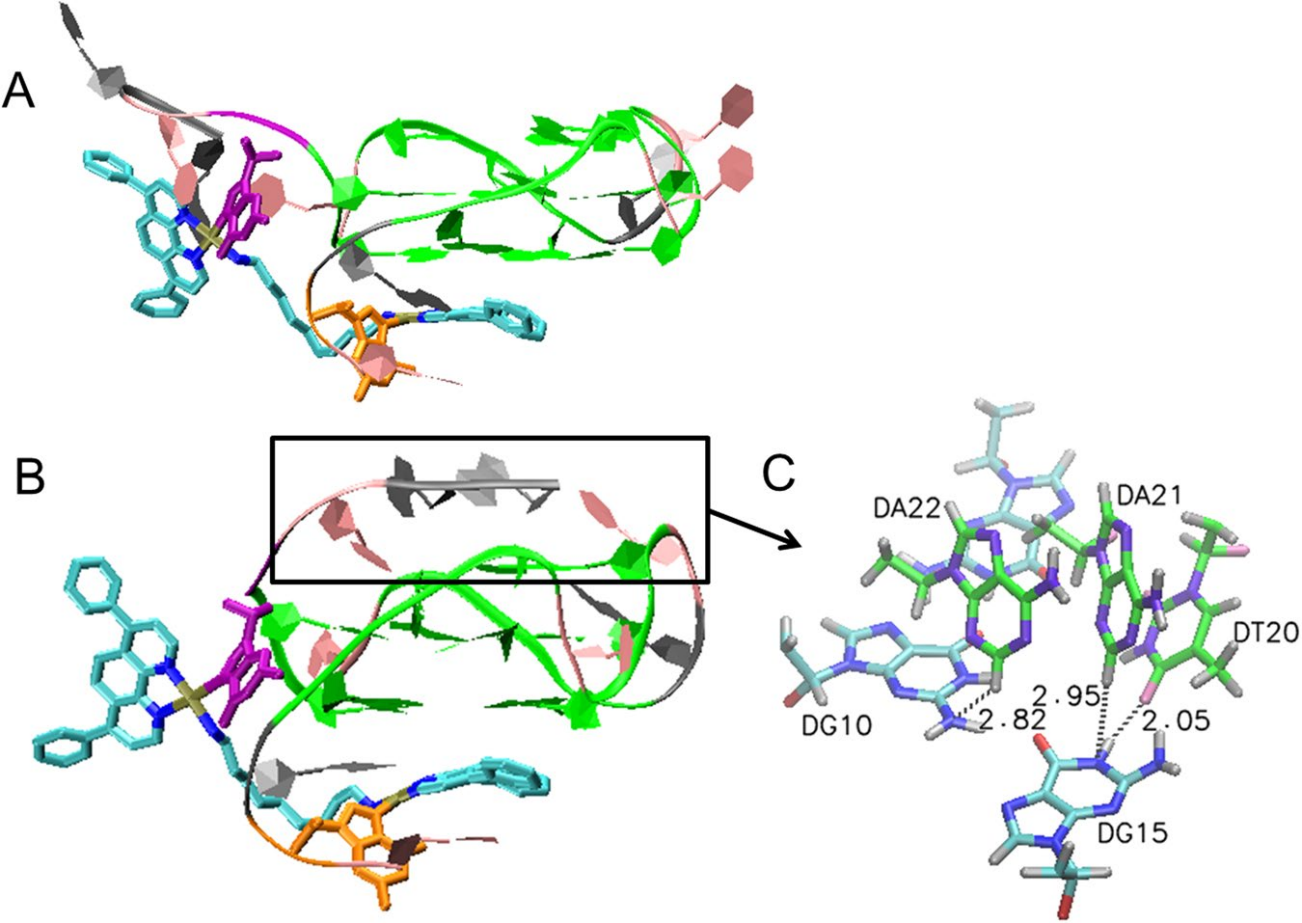
Two docking structures of dual cross-linked **Pt3**-GQ complex (**A**: conf1; **B**: conf2). **Pt3** and G_2_/G_19_ were shown in Licorice model (Pt: brown; N: blue; **C**: cyan; Cl: red; G_2_: orange; G_19_: purple), and the rest G-quadruplex was shown in ribbon model (T: pink; A: gray; G: green; G_2_: orange; G_19_: purple). (**C)** The hydrogen bond formed between 3′-A_22_A_21_T_20_ trinucleotides and top G-tetrad (G triad in cyan(C)/blue(N)/red(O); 3′-trinucelotides in green(C)/violet(N)/pink(O)).

For both docking conformations, a customized force field of the first cross-linking bond was calculated in MCPB.py, after the first cross-linking bond was tentatively formed between Pt(II) ion and N7 of G_2_ in Discovery studio 2016. The mono-crosslinking structure was then subjected to a position restraint MD simulation using GROMACS v4.6 (Figure S12)^32–34^ to optimize the conformation of mono-crosslinked **Pt3**-GQ adducts. As shown in Figure S13, two optimized mono-crosslinked conformations of **Pt3**-GQ adducts were yielded. Aromatic Dip of bottom Pt core remained well stacked with 5′-end G-tetrads, while G_19_ left the top G-tetrad to fall in the adjacent vicinity of the second Pt(Dip) core. The dissociation of 3′-end G-tetrad to G-triad also explained why the GQ stabilizing ability of **Pt3** was not so strong as **Pt0**_._ Hence, the force field of the second crosslinking bond can be built up between Pt(II) nucleus and N7 of G_19_ by MCPB.py. The structure of the dual crosslinking adduct was then submitted to REMD simulation against temperature fluctuation from 298 K to 331 K in order to explore a more stable conformation of the adducts. Two conformations with the highest cluster population were obtained (Fig. 4). In both conformations, one Pt(Dip) showed good stacking with 5′-end G-tetrad and cross-linked to G_2_ without significant disturbance to the bottom two layers of G-tetrads. Whereas, the second Pt(Dip) could approach G_19_ through the distinct orientations of the alkyl linkage and hence induced discrete conformation of 3′-end trinucleotides and top layer of G-triad, which could account for the different products after enzymatic cleavage of two dual cross-linking **Pt3**-GQ adducts by SVP. In Conf1, top Pt(Dip) approached G_19_ by crossing the backbone of G_17_ and pulled the 3′-end trinucleotides away from G-quadruplex upon the formation of cross-linking bond between Pt(II) and G_19_, which made 3′-AAT better accessible to exonuclease. On the contrary, in Conf 2, 3′-end trinucleotides looped back and formed H-bonds with the G-triad (Fig. 4C). Consequentially the interactions with the aromatic core of c-myc GQ made 3′-end of conf 2 more resistant to the enzymatic hydrolysis. Hence incomplete digestion products from 3′-end of **Pt3**-GQ adduct, **P2**, were observed in MS analysis.

### **Pt3**-c-myc GQ adducts efficiently inhibit the replication of c-myc sequence

Small molecular binders were often meant to stabilize GQ and consequently interfere the downstream biological processes, **Pt3** were not limited to stabilization effects, but can form covalent conjugation to G-rich sequence to achieve the same effects. A polymerase chain reaction stop (PCR-stop) assay was applied to a template containing G-rich c-myc sequence to show the efficient inhibition on the replication of c-myc sequence upon the formation of **Pt3**-GQ. The PCR reactions were performed in 1 × PCR buffer (20 mM Tris-HCl buffer, pH 8.4 with 50 mM KCl and 1.5 mM MgCl_2_). Figure S14 showed 53% inhibition on the extension of c-myc template occurred *via* forming Pt3-GQ adduct; while negligible inhibition occurred to the ht21 sequence due to the lack of cross-linking conjugation. This indicated that dual crosslinking bonds in **Pt3**-c-myc GQ adducts could contribute to the biological efficacy of **Pt3**, even when the thermal stability of the adduct as G-quadruplex may not be so ideal.

## Discussion

This series of dinuclear Pt complexes all possess dual functions at each Pt centre, with aromatic stacking with G-tetrads and cross-linking to purines in GQ sequence. Non-covalent π-π stacking between Dip ligand and 5′-end G-tetrad lead first Pt complex motif to the covalent chelating position near 5′-end purine, G_2_. With an optimal length and a proper binding in GQ grooves and loops, the alkyl chain further brought in the second Pt core to 3′-end of GQ sequence for the dual cross-linking. The unique structure and functionality endowed the dinuclear complex with specific reactivity to parallel GQ topology and to clamp c-myc GQ *via* dual covalent linkages, rather than the canonical single modal of π stacking. Both complex cores showed more efficient cross-linking to guanines than adenines, which resulted in the coordination not only to the loop guanine, G_2_, but also G_19_ in 3′-end G-tetrad. Though the thermal stability of GQ may be compromised by opening G-tetrad, the formation of covalent Pt-N bond at both sides of GQ sequences can prevent the genomic region from interacting enzymes and proteins much more thoroughly than the dynamic non-covalent binding of many GQ stabilizers without chemical reactivity. Whether the Pt-GQ adducts here would stay as GQ topology or not, irreversible impacts would be enforced on the downstream biological and pathogenic pathways to achieve higher anti-cancer therapeutic potency.

## Conclusion

In summary, a series of dinuclear platinum complexes were synthesized in this study. **Pt3** with a nonanediamine linkage showed the optimal stabilizing ability and selectivity on c-myc GQ. Further reacting dechlorinated Pt3 with c-myc GQ produced two dual cross-linking adducts with the chelation to the same guanines. Both HPLC analysis and theoretical modelling showed different conformations upon binding **Pt3** and the flexible alkyl linkage to c-myc GQ. Hence our results suggest that the dual functional dinuclear platinum complexes with appropriate bridging length could both stabilize and cross-link to GQ structure and offer a promising strategy in drug design of specific targeting guanine rich sequences in biological significant regions.

## Methods

### Materials

K_2_PtCl_4_ and 4,7-diphenyl-1,10-phenanthroline (Dip) were purchased from Alfa Aesar. 1,7-diaminoheptane, 1,8-diaminooctane, 1,9-diaminonoane, 1,10-diaminodecane, thiazole orange, and other salts were bought from Sigma-Aldrich.

### Synthesis and characterization of dinuclear platinum compounds

Pt(Dip)Cl_2_ was synthesized as previously report^25^. As a general procedure for synthesis of dinuclear platinum compounds, Pt(Dip)Cl_2_ was dissolved in dimethylformamide and stirred with 0.97 molar *eq* silver nitrate for 8 hours at room temperature. The silver chloride precipitation was removed through centrifuge; and half of the molar ratio of diamine was added to the supernatant and stirred for another 12 hours. The whole reaction was conducted at dark. The solution was reduced to 2 mL and the product was precipitated with 20 mL ethyl ether. The dinuclear platinum compounds was purified through silica gel with dichloromethane and methanol as elutes. Both ESI-MS and ^1^H-NMR were applied to characterize these compounds.

### Fluorescence melting studies

Double terminal DNA labeled with 6-FAM at 5′ and TAMRA at 3′ were chosen for FRET evaluation. F-c-myc-T DNA was diluted to 5 mM lithium cacodylate, pH 7.4, 50 mM LiCl and 1 mM KCl; and other DNA strands were diluted to 5 mM lithium cacodylate, pH 7.4, 50 mM LiCl, 5 mM KCl. The DNA solutions were heated to 95 °C for 5 minutes and then cooled to room temperature slowly. Competitive experiment was carried out with dual-fluorescent labeled DNA (F-c-myc-T, 0.2 μM), with **Pt3** (1.0 μM) and increasing amounts (0, 5, 15 and 50 equiv.) of unlabeled competitors ds26. Fluorescence melting curves were recorded on Roche Lightcycler 480 II real time PCR instrument with excitation at λ = 450–495 nm and detection at λ = 515–545 nm. 20 uL 0.2 uM DNA with 0.2 uM platinum compounds was applied to the wells of plate. Fluorescence data were collected over 37 °C to 95 °C with an interval of 1 °C.

### FID assays

FID data was collected on Varian Cary Eclipse fluorescence spectrophotometer. DNA was annealed in 10 mM lithium cacodylate buffer, pH 7.4, 100 mM KCl. The concentration for all DNAs was 0.25 μM, thiazole orange (TO) was 0.5 μM for G-quadruplex and 0.75 μM for ds26 DNA. Platinum compounds were titrated into DNA solution with increase concentration. Fluorescence spectra were recorded after each addition. The excitation wavelength is 485 nm and the TO displacement ratio is quantified by the decrease of fluorescence area.

### Reaction of Pt3 with DNA

**Pt3** (or the other three Pt complexes) was firstly dechlorinated with 4 molar *eq* silver nitrate for 12 h in DMF and then extracted with ethyl ether. Excess silver nitrate in aqueous solution was then removed by extraction over dichloromethane. After filtration, the filtrate was collected and used for the following reaction with DNA. DNA including ds26, ht21 and c-myc was annealed in 10 mM potassium phosphate buffer, pH 7.4, 100 mM KNO_3_ with concentration of 50 μM. After that, DNA was diluted to 10 μM to react with 20 uM pre-processed **Pt3** (or the other three Pt complexes). The reaction solution was subjected to HPLC for analysis. 300 µL of aliquots were injected onto an ChemcoPak column (5-ODS-H), with a size of 4.6 × 250 (W). Then the column was eluted with a 60-min linear gradient of 10–40% acetonitrile in 0.1 M triethylammonium acetate, pH 7.0. The flow rate was 1 mL/min and monitored with ultraviolet (*A*_260_) detector. The peaks were collected and analyzed with MALDI-TOF MS.

### MALDI-TOF MS determination of adducts of Pt3 with DNA

MALDI-TOF mass spectrometry analysis was performed on JMS-S3000 (JEOL, Japan). Matrix 3-hydroxy picolinic acid (3HPA, 10 mg) was dissolved in 200 μL 50% aqueous acetonitrile; diammonium citrate (DAC, 10 mg) was dissolved in 100 μL H_2_O; and then 3HPA was mixed with DAC with ratio of 8:1 (v/v). DNA and matrix were mixed with 1:1 volume ratio and then subjected onto plate for MS data record.

### Enzyme digestion of Pt3-GQ adducts

0.1 U SVP or 0.005 U BSP enzymes were applied to 200 pmol DNA/Pt adduct for 5 min at room temperature and then enzyme was deactivated though incubation at 95 °C for 5 mins. DNA in the solution was recovered by Ziptip C18 tips (Milipore, USA), and eluted by the matrix mixture for MALDI-TOF MS.

### Mass spectrum of rA-Pt3 and rG-Pt3 adducts

1 mM of rA, rG or rA/rG mixture reacted with 200 µM of **Pt3** in water for 24 h. The crude reaction mixture was then subjected to ESI mass spectrometry for analysis.

### Molecular docking of dual cross-linking Pt3-GQ conformations

*Constraint docking*: **Pt3** with 9 carbon linker showed the most significant stabilization effect to G-quadruplex and thus was used as the ligand in the simulation. The structure of **Pt3** was drawn in Gaussview (*Semichem Inc*., Shawnee Mission, KS) and optimized (B3LYP/6-31 G* for C,H,N,Cl; SDD for Pt) using Gaussian 09 (Gaussian, Inc., Wallingford CT). GOLD v5.4 (CCDC Software Limited) was used to dock binuclear Pt complex into the proximal position of c-myc G-quadruplex (PDB ID: 2L7V)^29^. To find the stable binding conformation of **Pt3** that potential can form dual crosslinking bond to G2 and G19 simultaneously, the distance constraint with the value of 5.0/Å^2^ was applied between Pt center and N7 of G2, and between the second Pt(II) and N7 of G19. The minimum separation for the constraint was set to be 3.0 Å and the maximum separation to be 6.0 Å. The docking center was set to be the center of the whole c-myc G-quadruplex and docking radius was set to be 30 Å to allow the software to explore all the possible binding sites of **Pt3** on c-myc GQ with two Pt centers close to G2 and G19, respectively. The scoring function was CHEMPLP and 30 runs were conducted to find the most stable binding conformation of Pt3 on c-myc GQ as the poses with the highest docking scores. The optimal docking conformation of **Pt3**-GQ were selected and visualized using VMD, (NIH Center for Macromolecular Modeling and Bioinformatics, at the Beckman Institute, University of Illinois at Urbana-Champaign), as shown in Figure S11^35^.

#### Setup of Pt-G force field

The force field for c-myc G-quadruplex apart from the chelating guanine was obtained from AMBER99bsc0. With the help of MCPB.py in Ambertools16, the force constant and atomic charge for the cross-linking guanines were calculated^36^. Simultaneously, the same parameters of all the coordination bonds on Pt(II) atom were calculated. The basic procedure was listed as followed. To build up a complete force field for a new molecule, the parameters for both bonded terms and non-bonded terms were determined. The non Pt-containing bonded terms were achieved from existing force filed: Amber99bsc0 force field was used for guanosine and GAFF force field was used for the rest ligands^37^. For the Pt-containing bonded terms, the equilibrium distance, angle and dihedral were computed as the corresponding value of optimized structure, which was obtained using Gaussian 09 software. The force constant was achieved from the Hessian matrices^38^. With regard to non-bonded terms, the atomic charge was evaluated using RESP method, the electrostatic potential information was computed by MK method in Gaussian 09 and then the atomic charge was fitted using RESP program in Ambertools^39,40^. The VDW parameters can be obtained readily from previous reference^41^. After that, some of the Pt containing terms were modified according to the reference^42^. The modified parameters were listed in Table S5 to S9 and each atom was label in Figure S12.

#### MD simulation for cross-linking **Pt3**-GQ conformation

To find the optimal conformation, the mono-crosslinked **Pt3**-GQ with customized Pt-G force field for the first Pt(II) and G2 was submitted to a PBC cubic box with the distance between solute and box side to be 1 nm, the box was then filled with water and neutralized with Na^+^. After the PBC cubic box was prepared by minimization, NVT (298 K, 100 ps) and NPT (1 atm, 100 ps) equilibriums sequentially, the system was then submitted to a 10 ns product MD with a distance restraint of 1.0 × 10^3^ kJ/(mol•nm^2^) applied between the second Pt(II) ion and N7 of G19. The same restraint was also applied to the heavy atoms of two bottom G-quartet (G5, G9, G14, G18, G4, G8, G13, G17) and bottom Dip ligand to maintain the binding and cross-linking conformation of one Pt(Dip) and G-quartets on the 5′-end of c-myc GQ. To form the second crosslinking bond, a customized force field was built up between Pt(II) ion and G19 at 3′-end of c-myc GQ. After submitted the dual cross-linked **Pt3**-GQ to a PBC cubic box as aforementioned, a new series of minimization, NVT (298 K, 100 ps), and NPT (1 atm, 100 ps) equilibrium were conducted. A 40 ns REMD simulation with 16 replicas in the presence of the same position restraint abovementioned was applied to the dual-crosslinked Pt3-GQ structure in GROMACS v5.1 package^43^. The temperature spanned from 298.00 K to 331.25 K (SI-3). The trajectory of replica at 298.00 K was then clustered and the cluster with the largest population was used to draw the conformation of dual-crosslinking **Pt3**-GQ complexes in Figure S13.

### PCR stop assay

The oligonucleotide c-myc (rev-c-myc) and ht21 (rev-ht21) were used here. c-myc and ht21 first reacted with 2 equiv. amount of **Pt3** for 24 hours. The PCR reactions were performed in 1× PCR buffer (20 mM Tris-HCl buffer, pH 8.4 with 50 mM KCl and 1.5 mM MgCl_2_), containing 8 pmol of each oligonucleotide, 16 pmol of **Pt3**, 0.2 mM dNTPs and 2.5 U Taq polymerase. Reaction mixtures were incubated in a Takara TP600 thermocycler with the following cycling conditions: 94 °C for 3 min, followed by 30 cycles of 94 °C for 30 s, 58 °C for 30 s, and 72 °C for 30 s. PCR products were then analyzed on 12% native polyacrylamide gels in 1× TBE and EB stained, then Typhoo scanner (Trio variable mode imager V5.0) was used for imaging.

## Electronic supplementary material

Supplementary Information
