# Supplementary material for: Dual functional dinuclear platinum complex with selective reactivity towards c-myc G-quadruplex
Source: Sci Rep. 2018 Jan 15;8:767. doi: 10.1038/s41598-017-19095-y (PMC5768759; doi:10.1038/s41598-017-19095-y)
Supplement: Supplementary file 1 — Supplementary Information [file 41598_2017_19095_MOESM1_ESM.pdf]

Supporting information for:

Dual functional dinuclear platinum complex with selective reactivity towards c-myc G-quadruplex

Lei He,[1a] Zhenyu Meng,[1a] Dechen Xu[1] and Fangwei Shao[1*]

[1]Division of Chemistry and Biological Chemistry, School of Physical and Mathematical Sciences, Nanyang Technological University, 21 Nanyang Link, Singapore 637371 (Singapore)

[a]These authors contributed equally to this work

*fwshao@ntu.edu.sg

Oligonucleotides sequence

All DNA was obtained from Sangon (Shanghai, China) with HPLC purification.

ds26: 5'-CAATCGGATCGAATTCGATCCGATTG-3'.

c-myc: 5'-TGAGGGTGGGTAGGGTGGGTAA-3'.

ht21: 5'-GGGTTAGGGTTAGGGTTAGGG-3'.

rev-c-myc: 5'-ATCGCTTCTCGTCTTACCCA-3'.

rev-ht21: 5'-TCTCGTCTTCCCTAA-3'.

F-ht21-T:5'-FAM-GGGTTAGGGTTAGGGTTAGGG-TAMRA-3'.

F-c-myc-T: 5'-FAM-TGGGGAGGGTGGGGAGGGTGGGGAAGG-TAMRA-3'.

F-bcl2-T: 5'-FAM-AGGGGCGGGCGCGGGAGGAAGGGGGCGGGAGCGGGGCTG-TAMRA-3'

F-c-kit-T: 5'-FAM-AGGGAGGGCGCTGGGAGGAGGG-TAMRA-3'.

F-duplex-T: 5'-FAM-TATAGCTATA-Spacer18-TATAGCTATA-TAMRA-3'. Spacer18 is an 18-atom hexa-ethyleneglycol spacer.

Characterization of dinuclear Pt complexes.

[{Pt(Dip)Cl}$_2$(μ-1,7-diaminoheptane)](NO$_3$)$_2$ (Pt1). Yield 70.4%, ESI-MS calc for [M^{2+}] m/z, 628.06, found 627.53. ^{1}H NMR 300 MHz (CD_2Cl_2): δ 9.89 (d, *J* = 5.79 Hz, 2H, H2), δ 9.73 (d, *J* = 5.64 Hz, 2H, H9), δ 8.08 (m, 6H, H3, H5 and H6), δ 7.87(d, *J* = 5.64 Hz, 2H, H8), δ 7.62 (m, 20H, Ph), δ 5.73 (bs, 4H, NH_2), δ 3.14 (s, 4H, CH_2), δ 1.95 (s, 4H, CH_2), δ 1.48 (s, 6H, CH_2).

[{Pt(Dip)Cl}$_2$(μ-1,8-diaminooctane)](NO$_3$)$_2$ (Pt2). Yield 62%, ESI-MS calc for [M^{2+}] m/z, 635.08, found 634.55. ^{1}H NMR 300 MHz (CD_2Cl_2): δ 9.84 (d, *J* = 5.67 Hz, 2H, H2), δ 9.77 (d, *J* = 5.67 Hz, 2H, H9), δ 8.08 (m, 6H, H3, H5 and H6), δ 7.90(d, *J* = 5.67 Hz, 2H, H8), δ 7.61 (m, 20H, Ph), δ 5.74 (bs, 4H, NH_2), δ 3.14 (s, 4H, CH_2), δ 1.95 (s, 4H, CH_2), δ 1.32 (s, 8H, CH_2).

[{Pt(Dip)Cl}$_2$(μ-1,9-diaminonoane)](NO$_3$)$_2$ (Pt3). Yield 71.3%, ESI-MS calc for [M^{2+}] m/z, 642.09, found 642.65. ^{1}H NMR 300 MHz (CD_2Cl_2): δ 9.93 (d, *J* = 5.64 Hz, 2H, H2), δ 9.79 (d, *J* = 5.67 Hz, 2H, H9), δ 8.12 (m, 6H, H3, H5 and H6), δ 7.93(d, *J* = 5.67 Hz, 2H, H8), δ 7.65 (m, 20H, Ph), δ 5.80 (bs, 4H, NH_2), δ 3.17 (s, 4H, CH_2), δ 2.01(s, 4H, CH_2), δ 1.40 (s, 10H, CH_2).

[{Pt(Dip)Cl}$_2$(μ-1,10-diaminodecane)](NO$_3$)$_2$ (Pt4). Yield 56.7%, ESI-MS calc for [M^{2+}] m/z, 649.10, found 649.22. ^{1}H NMR 300 MHz (CD_2Cl_2): δ 9.90 (d, *J* = 5.67 Hz, 2H, H2), δ 9.71 (d, *J* = 5.67 Hz, 2H, H9), δ 8.07 (m, 6H, H3, H5 and H6), δ 7.88(d, *J* = 5.67 Hz, 2H, H8), δ 7.61 (m, 20H, Ph), δ 5.79 (bs, 4H, NH_2), δ 3.12 (s, 4H, CH_2), δ 1.93(s, 4H, CH_2), δ 1.41 (s, 12H, CH_2).

Table S1. Stabilization temperature ΔT_m of different DNA structures in the presences of platinum compounds from FRET-based thermal melting.

	ΔT_m (°C)				
	**Pt1**	**Pt2**	**Pt3**	**Pt4**	**Pt0**
F-c-myc-T	2.6	3.2	8.5	3.0	11.5
F-bcl2-T	0.6	0.6	0.8	0.4	5.0
F-c-kit-T	0.7	1.1	0.6	1.4	9.6
F-ht21-T	0.9	1.1	1.5	1.7	3.2
F-duplex-T	-0.5	-0.2	-0.2	-0.01	-0.4

Table S2. DC_{50} (the concentration of **Pt3** needed to displace 50% of thiazole orange (TO)) values for different DNA.

**DNA**	**DC_{50} (μM)**
c-myc	0.20
ht21	1.17
ds26	1.84

Table S3. Assignment for the peaks on MALDI-TOF MS spectra of peaks on HPLC of ds26 and **Pt3**-ds26 adducts.

**Peak**	**Composition**	**Formula**	**obsd**	**cald.**
D1	ds26	C254H321N97O154P25	7972.69	7971.24
D2	ds26	C254H321N97O154P25	7971.72	7971.24
D3	ds26	C254H321N97O154P25	7970.32	7971.24
	ds26+$Pt_2(Dip)_2$(diaminonoane)	C311H371N103O154P25Pt2	9182.54	9180.46
D4	ds26+$Pt_2(Dip)_2$(diaminonoane)	C311H371N103O154P25Pt2	9181.64	9180.46

Table S4. Assignment of MALDI-TOF MS spectra for peaks on HPLC of c-myc and **Pt3**-GQ adducts.

**Peak**	**Composition**	**Formula**	**obsd**	**cald**
c-myc	c-myc	C220H270N95O131P21	6991.30	6992.57
P1	c-myc+$Pt_2(Dip)_2$(diaminonoane)	C277H320N101O131P21Pt2	8200.74	8201.80
P2	c-myc+$Pt_2(Dip)_2$(diaminonoane)	C277H320N101O131P21Pt2	8201.17	8201.80

Table S5. The modified bond terms for Guanine-Pt complex

Bond	R_0	K_r (kJ/(nm^2*mol))
**N7-Pt**	2.01	1.5313×10^5

Table S6. The modified angle terms for Guanine-Pt complex

Angle	θ_0	K_θ (kJ/(degree*mol))
**N1'-Pt-N2'**	90	1.6688×10^3
**N1'-Pt-N3'**	180	1.6044×10^3
**N2'-Pt-N3'**	90	1.5382×10^3
**Pt-N7-C8**	127.95	1.0385×10^3
**Pt-N7-C5**	127.95	1.3339×10^3
**N1'-Pt-N7**	90	1.3551×10^3
**N2'-Pt-N7**	180	1.4041×10^3
**N3'-Pt-N7**	90	1.4495×10^3

Table S7. The modified dihedral terms for Guanine-Pt complex

Torsional angle	φ_0	Vn(kJ/mol)	n
**C5-C7-Pt-N1'**	90	2.09	2
**C8-C7-Pt-N1'**	90	2.09	2
**C5-C7-Pt-N2'**	90	2.09	2
**C8-C7-Pt-N2'**	90	2.09	2
**C5-C7-Pt-N3'**	90	2.09	2
**C8-C7-Pt-N3'**	90	2.09	2

Table S8 The modified improper torsional terms for Guanine-Pt complex

Improper angle	χ_0	K_χ(kJ/mol)
**C8-C7-C5-Pt**	180	41.8

Table S9 The modified atomic charge for Guanine-Pt complex

Atom	Atomic charge
**N9**	-0.019
**C8**	0.3057
**H8**	0.069
**N7**	-0.3433
**C5**	0.009
**C6**	0.6837
**O6**	-0.5699
**N1**	-0.5053
**H1**	0.352
**C2**	0.7432
**N2**	-0.923
**H21 and H22**	0.4235
**N3**	-0.6636
**C4**	0.3847
**N1’**	-0.11232
**N2’**	-0.13805
**N3’**	-0.41289

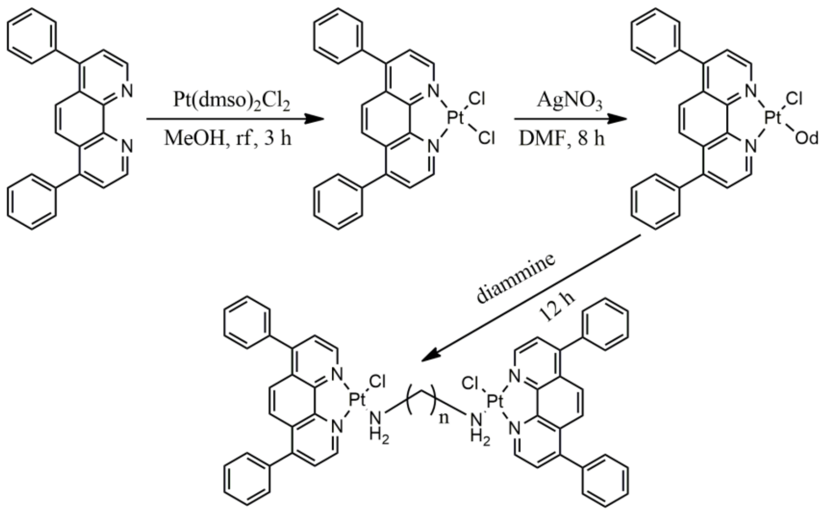

Scheme S1. Synthetic route of dinuclear platinum compounds, n = 7 ~ 10.

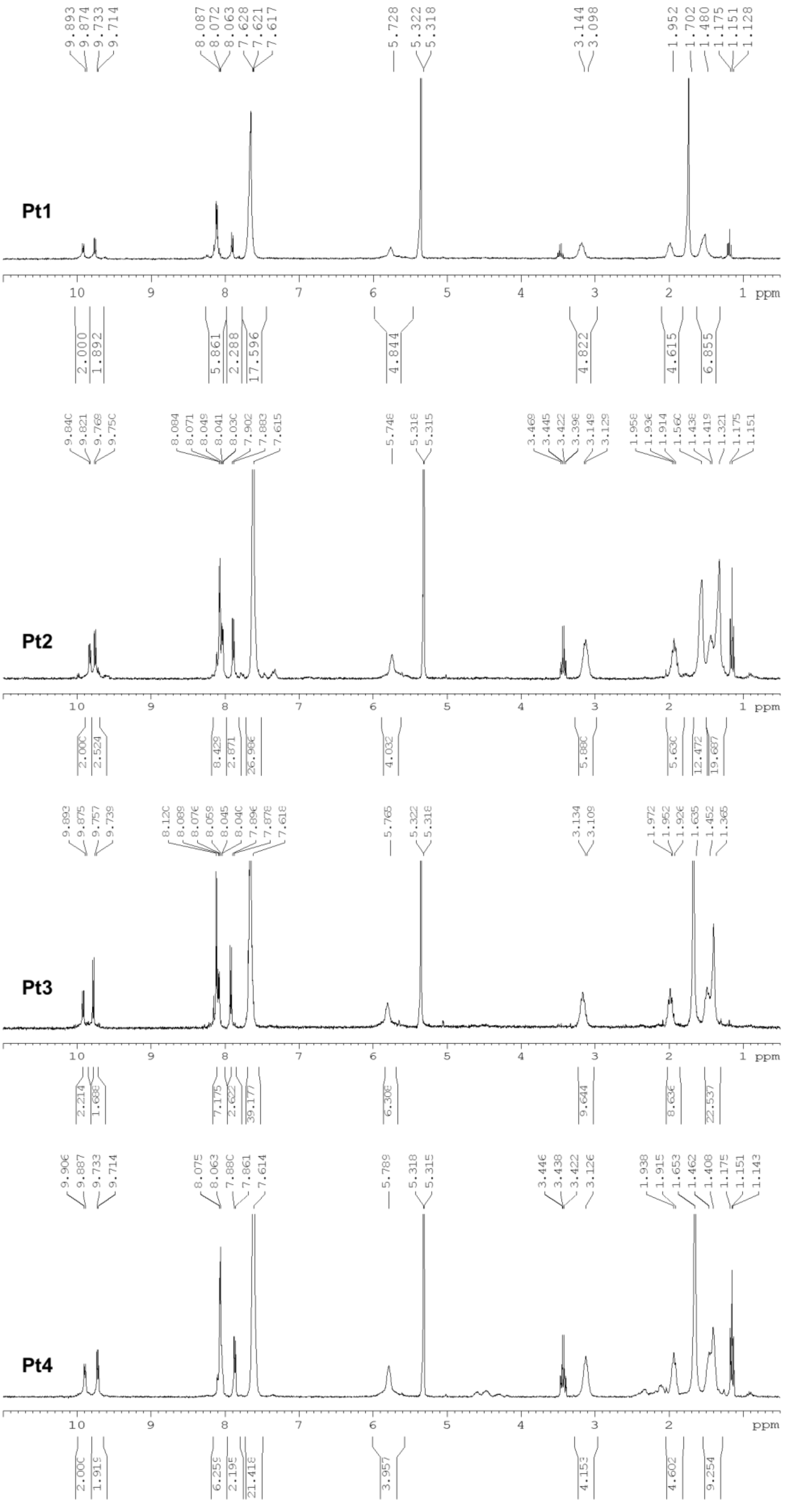

Figure S1. ^{1}H NMR spectrum of **Pt1-4** in CD_2Cl_2.

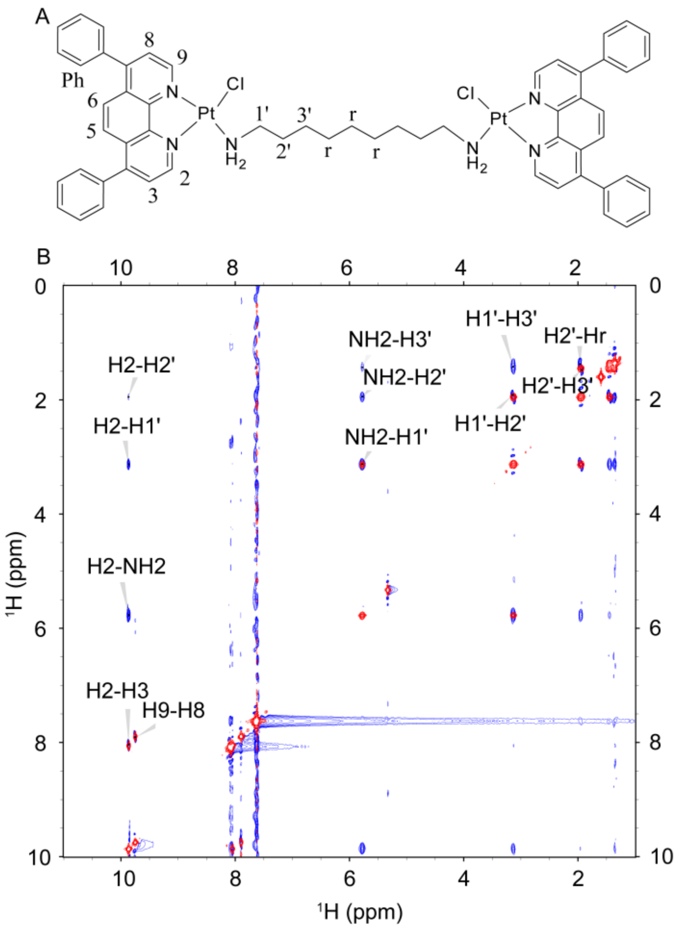

Figure S2. Overlay of ^{1}H-^{1}H COSY (red) and ROESY (blue) spectrum of **Pt3** in CD_2Cl_2.

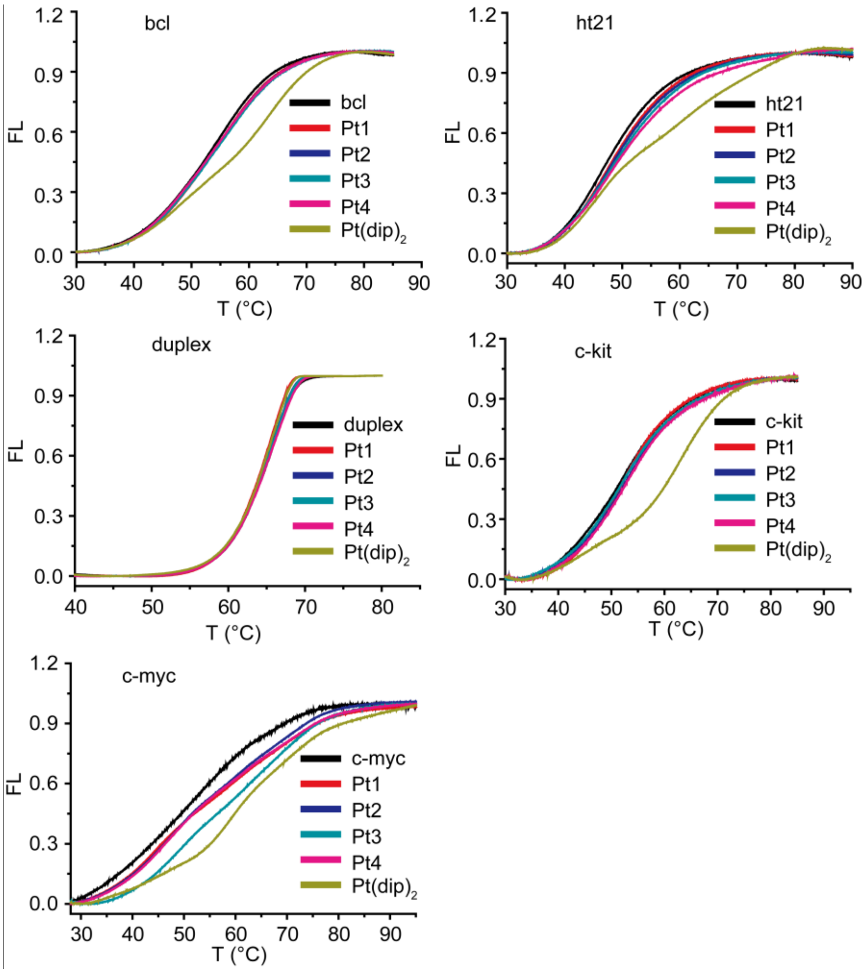

Figure S3. FRET melting profiles for 200 nM DNA without or with 200 nM different platinum compounds, in 10 mM lithium cacodylate buffer, pH 7.4, 50 mM LiCl, 5 mM KCl.

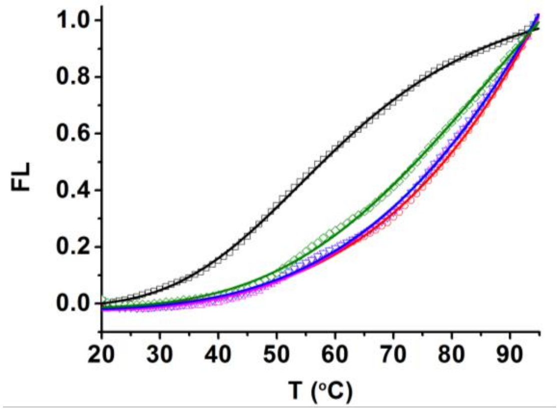

Figure S4. Competitive FRET-melting of 200 nM F-c-myc-T (black) with 1.0 µM **Pt3** and increasing amounts (0 [red], 5 [magenta] 15 [blue] and 50 [olive] equiv.) of unlabeled competitors (ds26).

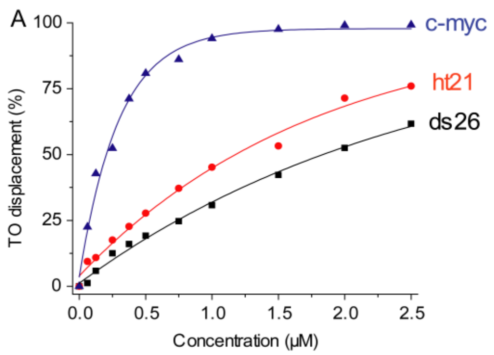

Figure S5. Plot of thiazole orange (TO) displacement *vs.* concentrations of **Pt3** with different DNAs.

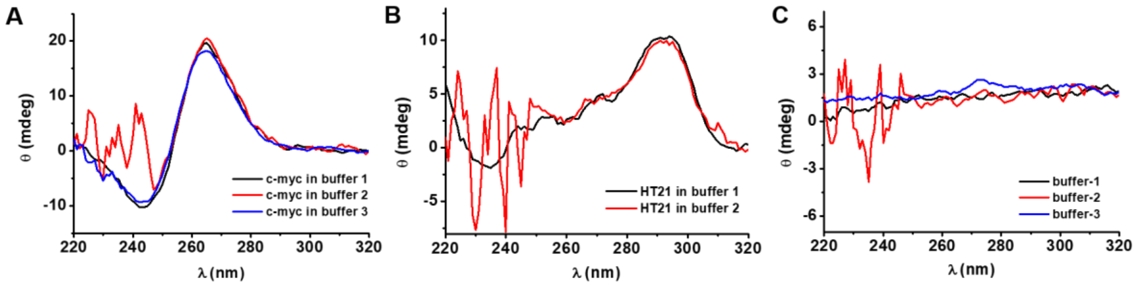

Figure S6. CD spectra of c-myc (A) and ht21 (B) in different buffers. Buffer background in CD spectra were shown in (C). **Buffer 1** is 5 mM lithium cacodylate buffer (pH 7.4) with 50 mM LiCl and 1 mM (for c-myc) or 5 mM (for ht21) KCl; **buffer 2** is 10 mM potassium phosphate buffer (pH 7.4) with 100 mM KNO_3; **buffer 3** is 1 × PCR buffer – 20 mM Tris-HCl (pH 8.4) with 50 mM KCl and 1.5 mM $MgCl_2$.

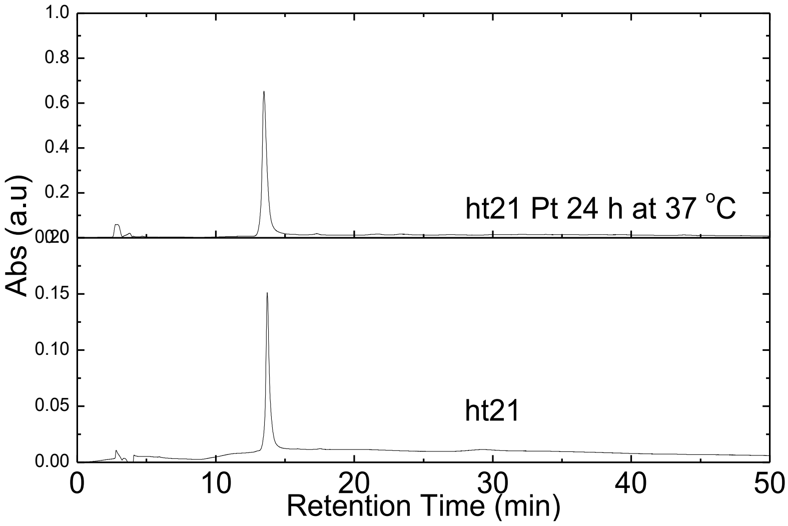

Figure S7. HPLC profiles of 10 μM ht21 G-quadruplex with or without 20 μM **Pt3**.

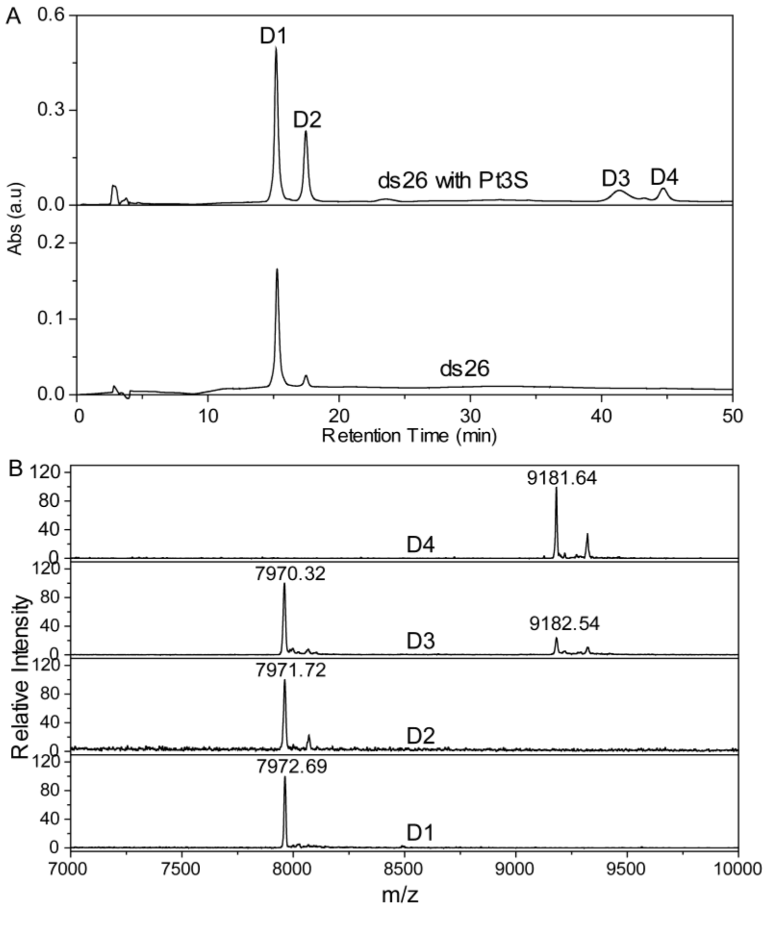

Figure S8. HPLC chromagraphy of ds26, and 10 μM ds26 duplex reacted with 20 μM **Pt3** for 24 hours at 37 °C (A); MALDI-TOF MS spectra of peaks from HPLC (B).

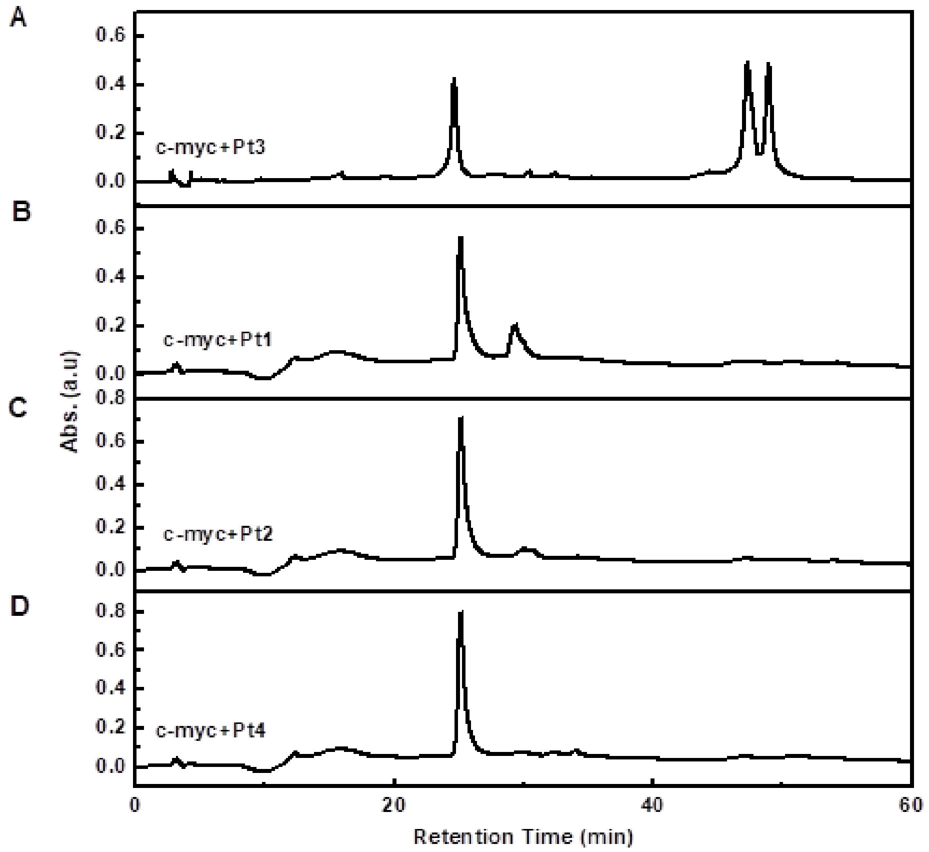

Figure S9. HPLC chromagraphy of c-myc, and 10 μM c-myc reacted with 20 μM **Pt3** (A), **Pt1** (B), **Pt2** (C) and **Pt4** (D) for 24 hours at 37 ^{o}C.

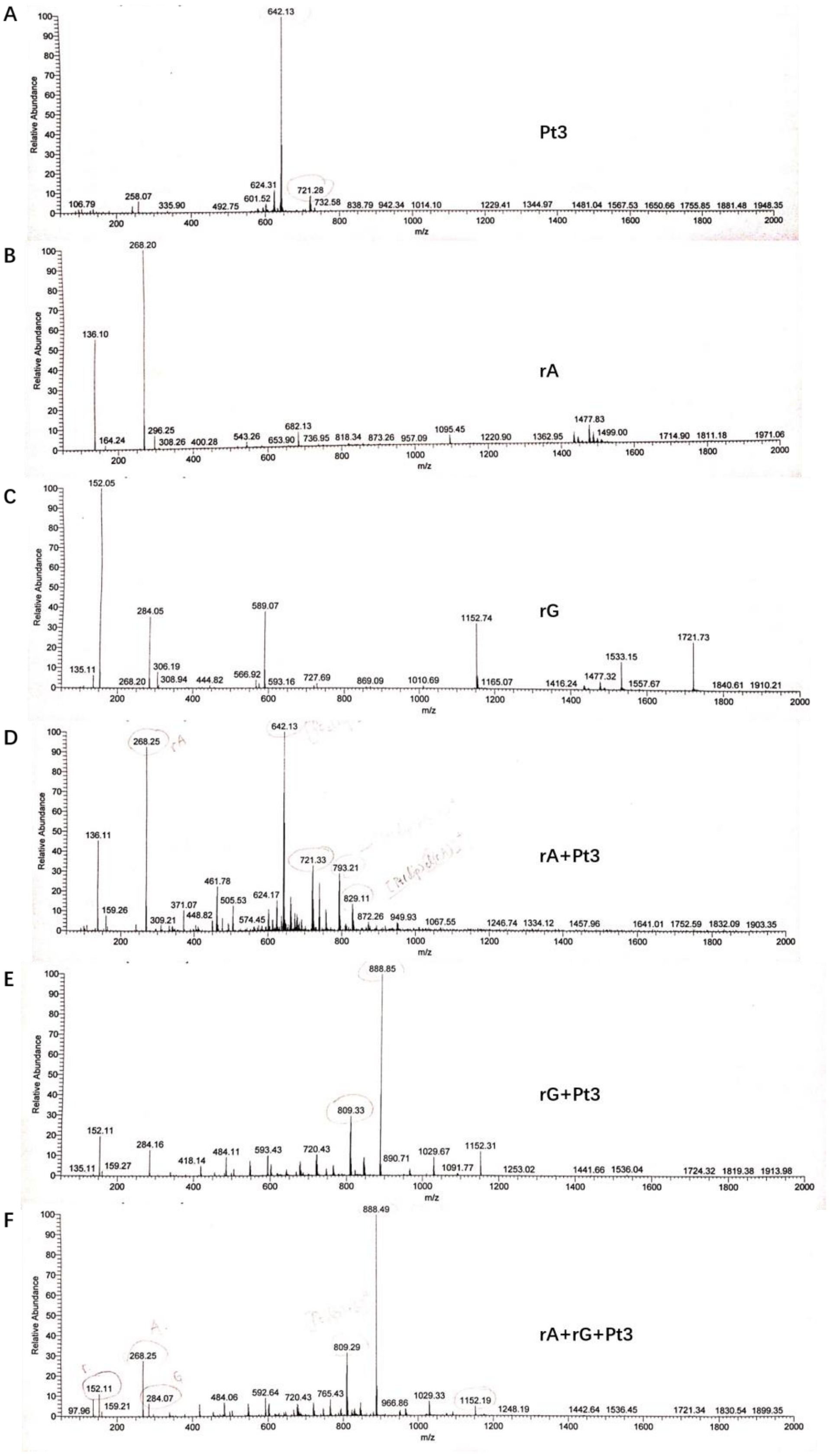

Figure S10. Mass spectrum of **Pt3** (A), adenosine (B), guanosine (C) and the adducts after 24-hour reaction. Adenosine with **Pt3** (D), guanosine with **Pt3** (E), and adenosine plus guanosine with **Pt3** (F).

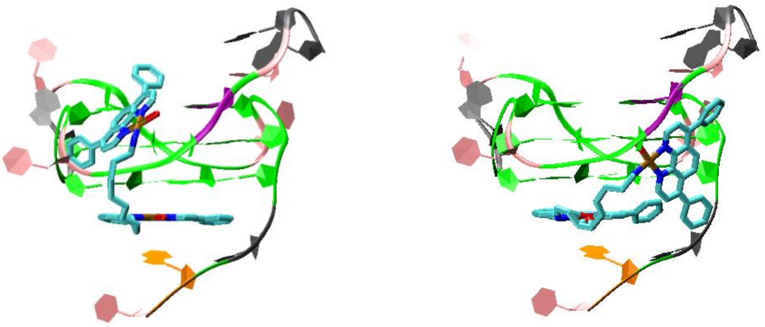

Figure S11. Molecular docking of **Pt3** to c-myc G-quadruplex. Left: Conf1; right: Conf2. The G-quadruplex was represented by new ribbon method (guanine: green; adenine: gray; thymine; pink; G2: orange; G19: pruple). Complex **Pt3** was presented in Licorice method (Pt: yellow; N: navy blue; Cl: red; C: blue).

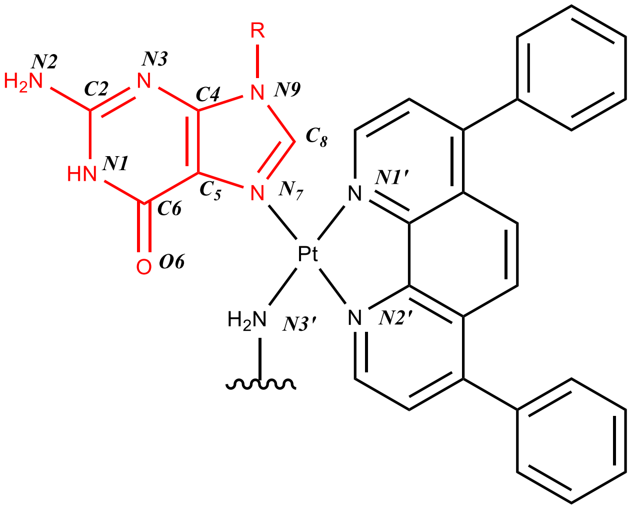

Figure S12. Structure of Pt-G complex. The atom types of coordinate atoms and guanine atoms are also labeled.

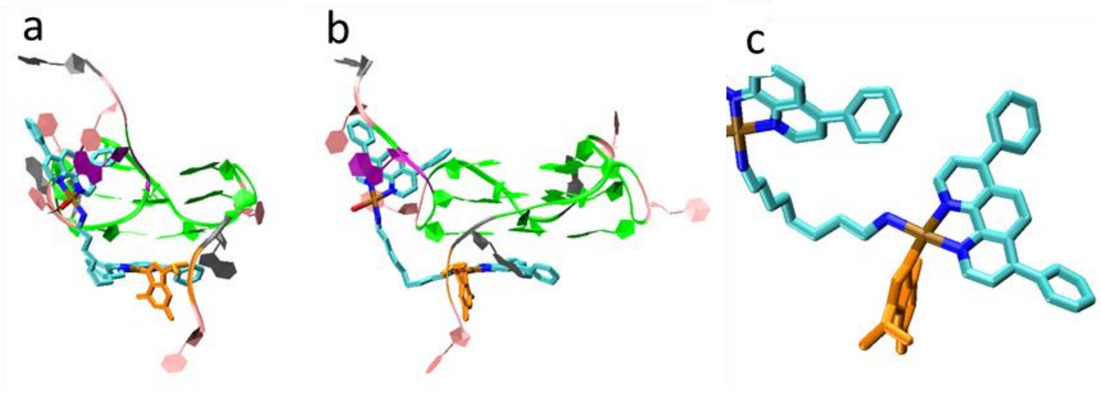

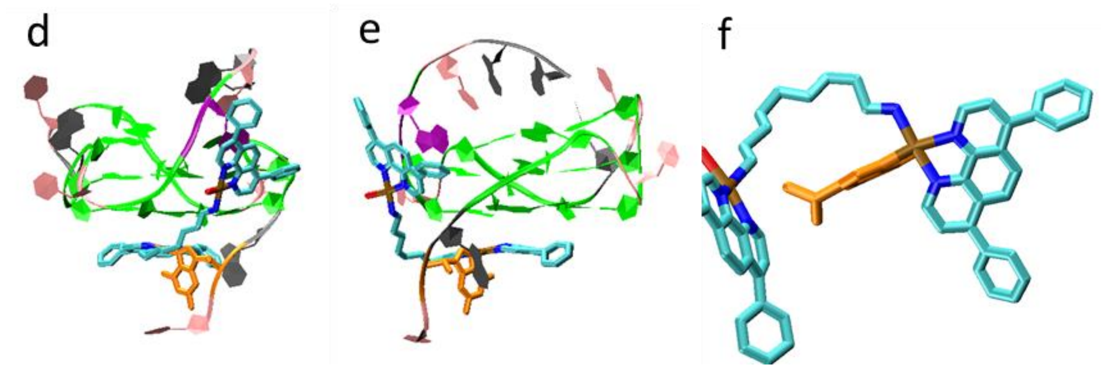

Figure S13. The structure of the two mono-crosslinked **Pt3** to c-myc GQ after 10 ns MD (a~c: Conf1; d~e: Conf2). For both conformations, the top Pt planes were close to G19 (a,d), another side view showed the top G-quartet has already decomposed under restraint (b,e). Zoom in of bottom Pt-G2 complex showed that the planar coordination geometry of Pt core has formed and G2 was nearly perpendicular to Dip ligand (c,f). The Pt complex and G2 were represented using Licorice model (Pt: brown; N: blue; C: cyan; Cl: red; guanine: orange), and the rest G-quadruplex was represented by new ribbon model (T: pink; A: gray; G: green; G2: orange; G19: purple).

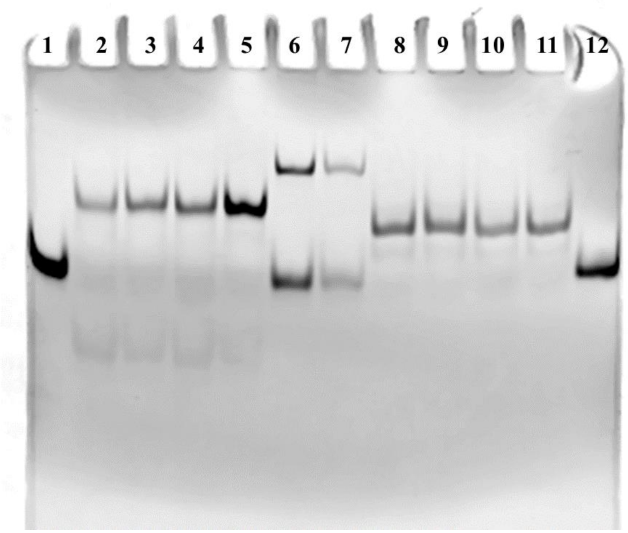

Figure S14. Inhibitory effects of **Pt3** on c-myc and ht21 templates extension in PCR-stop assay. Lane 1 and 12: control of duplex DNA – ds26; lane 6 and 7: control of both 21- and 48-mer duplex DNA; lane 5: G-rich (c-myc) sequence without **Pt3**; lane 2-4: three repeats of G-rich (c-myc) sequence with 2 equiv. amount of **Pt3**; lane 8: G-rich (ht21) sequence without **Pt3**; lane 9-11: three repeats of G-rich (ht21) sequence with 2 equiv. amount of **Pt3**.
